# HPLC-Fingerprints and Antioxidant Constituents of *Phyla nodiflora*

**DOI:** 10.1155/2014/528653

**Published:** 2014-07-20

**Authors:** Fang-Ju Lin, Feng-Lin Yen, Pei-Chun Chen, Moo-Chin Wang, Chun-Nan Lin, Chiang-Wen Lee, Horng-Huey Ko

**Affiliations:** ^1^Department of Fragrance and Cosmetic Science, College of Pharmacy, Kaohsiung Medical University, Kaohsiung 807, Taiwan; ^2^Institute of Biomedical Sciences, Sun Yat-Sen University, Kaohsiung 804, Taiwan; ^3^Graduate School of Nursing, Division of Basic Medical Sciences and Chronic Disease and Health Promotion Research Center, Chang Gung University of Science and Technology, Chiayi 613, Taiwan; ^4^Research Center for Industry of Human Ecology, Chang Gung University of Science and Technology, Taoyuan 333, Taiwan

## Abstract

*Phyla nodiflora* is a creeping perennial herb, widely distributed in the most tropical and subtropical regions. It has been used as a folk medicine, herbal beverage, or folk cosmetic. For these usages, the development of a chemical quality control method of this plant is necessary. In the present study, ten compounds, namely, 3,7,4′,5′-tetrahydroxy-3′-methoxyflavone (**1**), nodifloretin (**2**), 4′-hydroxywogonin (**3**), onopordin (**4**), cirsiliol (**5**), 5,7,8,4′-tetrahydroxy-3′-methoxyflavone (**6**), eupafolin (**7**), hispidulin (**8**), larycitrin (**9**), and *β*-sitosterol were isolated from the methanolic extract of the aerial part of *P. nodiflora* (PNM) and their structures were identified by 1D-NMR comparing their spectra with the literature. The antioxidant activities of these compounds were evaluated by free radical scavenging activity and tyrosinase inhibitory effect in cell-free systems. Compounds **4**, **5**, and **7** showed strong antioxidant activity. To control the quality of *P. nodiflora*, a simple and reliable method of high-performance liquid chromatography combined with ultraviolet detector (HPLC-UV) was established for both the fingerprint analysis and the quantitative determination of two selected active compounds, onopordin (**4**) and eupafolin (**7**). Statistical analysis of the obtained data demonstrated that our method achieved the desired linearity, precision, and accuracy. The results indicated that the developed method can be used as a quality evaluation method for PNM.

## 1. Introduction

*Phyla nodiflora* (L.) Greene (syn.* Lippia nodiflora* (L.) Michx, Verbenaceae), a creeping perennial herb with small white flowers, is distributed throughout India, Ceylon, Bangladesh, Baluchistan, Africa, and most tropical and subtropical regions [1]. In Taiwan,* P. nodiflora* has been used as a folk medicine in the form of a herbal drink, nourishing agent, immunomodulator, and as an anti-inflammatory agent [2].* P. nodiflora* possesses many pharmacological activities such as anti-inflammatory, analgesic, antipyretic, antiatherosclerotic, antidandruff, antibacterial, hepatoprotective, antiurolithiatic, antimicrobial, and antioxidant abilities [3–7]. In a previous study, we demonstrated that the methanolic extract of the aerial part of* P. nodiflora* (PNM) exerted an antimelanogenesis effect by downregulating the microphthalmia-associated transcription factor (MITF) expression level and decreasing the tyrosinase activity and melanin production [8]. Abbasi et al. [9] also mentioned the ethnopharmacological application of* P. nodiflora* for skin diseases and in folk cosmetics, for the treatment of pimples, carbuncles, and skin burns.

Previous phytochemical studies on this plant have afforded flavonoids, quinols and quinol glucosides, steroids, phenylpropanoids, alkaloids, resin, tannins, terpenoids, and volatiles [10–13]. It is well known that the majority of pharmacological effects of medicinal herbs could be attributed to their secondary metabolites. However, various factors, such as different cultivation areas, climatic conditions, and harvestable seasons, may significantly affect the level of these components. Thus, a systematic quality standard for quality assessment is imperative. In fact, no HPLC method was established for analysis of this herbal medicine; therefore, developing a suitable quality control method for it is required.

Based on preliminary screening data, PNM showed a strong radical scavenging activity and antimelanogenesis effect. These findings led us to focus on the isolation of active components in PNM; meanwhile, a method combined with high-performance liquid chromatography (HPLC) with ultraviolet (UV) detector was developed for the simultaneous chemical fingerprint and quantification of the active components. The results indicated that PNM possesses good antioxidant and antityrosinase potentials and the developed fingerprint could further serve for quality and quantity analysis of PNM added in cosmetic industry and herbal medicines.

## 2. Materials and Methods

### 2.1. General

Melting points were recorded on an electrothermal MEL-TEMP 3.0 apparatus. UV spectra were measured in methanol on a Beckman Coulter-DU 800 UV-visible spectrophotometer. IR spectra were recorded on a Perkin Elmer system 2000 FT-IR spectrophotometer. ^1^H and ^13^C NMR spectra were measured and recorded on a Bruker-400 MHz FT-NMR spectrometer and a Mercury-400 MHz FT-NMR spectrometer. ESI-MS were recorded on a Bruker Daltonics Apex II 30e. The absorbances in bioassays were measured and recorded on a multiplate spectrophotometer (*μ*Quant, BioTek, USA).

### 2.2. Plant Material, Extraction, and Isolation

*P. nodiflora* was collected in June 2010 in Tainan, Taiwan, and identified by Professor I. S. Chen, School of Pharmacy of Kaohsiung Medical University, Kaohsiung, Taiwan. A voucher specimen (2010-06-PNM) was deposited at the Herbarium of the Department of Fragrance and Cosmetic Science, Kaohsiung Medical University, Kaohsiung, Taiwan. The dried aerial part of* P. nodiflora* (4.6 kg) was chopped and immersed in methanol for three times at room temperature. The mixtures were filtered and concentrated to dryness under reduced pressure, producing a methanolic extract (PNM, 525 g). The PNM (160 g) was taken and further purified with* n*-hexane and an increasing polarity of EtOAc and MeOH, yielding 8 fractions (Fr. D_1_–D_8_). *β*-Sitosterol and 3,7,4′,5′-tetrahydroxy-3′-methoxyflavone (**1**) (3.9 mg) were purified by silica column chromatography (CC) with* n*-hexane : acetone (10 : 1), followed by* n*-hexane : CH_2_Cl_2_ : EtOAc (5 : 2 : 1) from Fr. D_2_. Hispidulin (**8**) (4.3 mg) and larycitrin (**9**) (1.2 mg) were purified by silica CC with* n*-hexane : EtOAC (2 : 3), followed by RP18 silica gel with acetone : H_2_O (3 : 2) from Fr. D_4_. Nodifloretin (**2**) (9.5 mg) and eupafolin (**7**) (13.5 mg) were isolated and purified by silica gel with* n*-hexane : EtOAC : MeOH (7 : 80 : 13), followed by RP18 silica with acetone : H_2_O (1 : 1) and Sephadex LH-20 CC from Fr. D_5_. 4′-Hydroxywogonin (**3**) (10.4 mg), onopordin (**4**) (21.8 mg), cirsiliol (**5**) (11.8 mg), and 5,7,8,4′-tetrahydroxy-3′-methoxyflavone (**6**) (10.3 mg) were isolated and purified by silica gel with* n*-hexane : EtOAC : MeOH (6 : 69 : 25), followed by RP18 gel with MeOH : H_2_O (1 : 1), acetone : H_2_O (3 : 1), and Sephadex LH-20 CC from Fr. D_6_. Compounds** 1**–**9** were recrystallized and identified by NMR and other spectroscopic data following comparison with values in the literature [11, 13, 14].

Another two batches of* P. nodiflora *were also collected at the same place in different years and seasons as shown in specimen numbers 2007-02-PNM and 2009-06-PNM. 2007-02-PNM was collected in February (spring) 2007 and 2009-06-PNM was collected in June (summer) 2009, respectively, and they were kept at our laboratory for future reference. Together with 2010-06-PNM, the air-dried samples of these three batches were lyophilized and stored at low temperature until analysis.

### 2.3. Chemicals and Reagents

2,2′-Azino-bis(3-ethylbenzothiazoline-6-sulfonic acid) diammonium salt (ABTS), 2,2-diphenyl-1-picrylhydrazyl (DPPH), ethylenediaminetetraacetic acid disodium salt dehydrate (EDTA∙2Na), nitrotetrazolium blue chloride (NBT), L-tyrosine disodium salts, tyrosinase (from mushroom, 5370 U/mg), xanthine, and xanthine oxidase (from buttermilk, 0.07 U/mg) were all purchased from Sigma-Aldrich (St. Louis, MO, USA). L-Ascorbic acid was obtained from J. T. Baker (Austin, USA). All chemicals and solvents were of high-performance liquid chromatography (HPLC) or analytical grade. Ethanol (99.8%) was purchased from Panreac (Barcelona, Spain). HPLC-grade methanol was obtained from ECHO (Miaoli, Taiwan). Sodium phosphate buffer (PBS) was freshly prepared in our laboratory at different concentrations and adjusted to the appropriate pH value before use. Ultrapure water was used in the experiments (Milli-Q system, Millipore, Massachusetts, Molsheim, France).

### 2.4. DPPH Radical (DPPH^∙^) and ABTS Cation Radical (ABTS^∙+^) Scavenging Assay

In the DPPH radical (DPPH^∙^) scavenging assay [15], fifty microliters of samples of various concentrations were mixed with 150 *μ*l of DPPH^∙^ solution (0.1 mM) dissolved in methanol. Each mixture was allowed to stand for 30 min and measured at 517 nm using a multiplate spectrophotometer (*μ*Quant, Bio-Tek, USA). The scavenging ability was calculated using the following formula:

(1)  Scavenging  ability  (%) =(1−sample absorbancecontrol absorbance)×100%.

The ABTS decolorization assay was performed as described [16], with slight modification. The ABTS cation radical (ABTS^∙+^) was produced by reacting 7 mM stock solution of ABTS with 2.45 mM potassium persulfate (final concentration) and allowing the mixture to stand in the dark for 6–8 h at room temperature before use. The ABTS^∙+^ solution was diluted to an absorbance of 0.7 ± 0.02 at 734 nm. Thirty microliters of samples of different concentrations were mixed with 170 *μ*l of ABTS^∙+^ working solution under a dark condition and shaken for 15 s. The absorbance was measured at 734 nm after 20 min. The scavenging capacity was calculated using the same formula as for the DPPH radical scavenging assay.

### 2.5. Superoxide Anion Radical (O_2_^∙−^) Scavenging Assay and Mushroom Tyrosinase Inhibition Assay

The procedures of O_2_^∙−^ scavenging activity and mushroom tyrosinase inhibitory effect were performed as previous report [17]. In the O_2_^∙−^ scavenging assay, xanthine (0.1 mM, 15 *μ*l) in 50 mM phosphate buffer (pH 7.4), NBT (25 *μ*M, 16 *μ*l), EDTA (0.1 *μ*M, 14 *μ*l), and 100 *μ*l of various concentrations of test compounds were mixed in the 96-well plates. Reactions were initiated by adding 0.5 U xanthine oxidase. After 30 min, the absorbance of each mixture was measured at 540 nm. In the mushroom tyrosinase inhibitory assay, L-tyrosine (2 mM, 80 *μ*l) in 50 mM phosphate buffer (pH 6.5) and various concentrations of test compounds (100 *μ*l) were added to 96-well plates. After 10 min incubation, 20 U mushroom tyrosinase was added to start the reaction. The absorbance of each mixture was measured at 490 nm after 30 min incubation. The O_2_^∙−^ scavenging capacity and the percentage inhibition of mushroom tyrosinase were calculated using the same formula as for the DPPH radical scavenging assay.

### 2.6. Lineweaver-Burk Plots

This study used Lineweaver-Burk plots to determine the inhibitory mode of test samples [15]. The kinetic study was performed in the presence or absence of test samples with various concentrations of tyrosine or xanthine as the substrate.

### 2.7. HPLC Conditions

Chromatographic analysis was performed using an ELITE LaChrom high- performance liquid chromatography (HPLC) system coupled with a L-2420 UV-Vis detector, a L-2200 autosampler, and a L-2130 pump. Chromatographic data were processed by Hitachi Model D-2000 Elite chromatography data station software. The chromatographic separations were performed on a Hypersil ODS-C18 column (250 mm × 4.6 mm i.d., 5 *μ*m). The mobile phase consisted of 0.1% formic acid (FA) and MeOH (M), and the flow rate was 0.5 ml/min. The gradient elution program was conducted as follows: a gradual increase of M 0–2% (0–5 min), M 2–6% (5–9 min), M 6–8% (9–12 min), M 8–12% (12–14 min), M 12–17% (14–20 min), M 17–22% (20–30 min), M 22–27% (30–35 min), M 27–31% (35–41 min), M 31–40% (41–60 min), M 40–43% (60–75 min), M 43–45% (75–80 min), and M 45–80% (80–120 min). The wavelength of the detector was set at 326 nm and the sample injection volume was 20 *μ*l.

### 2.8. Preparation of Standard Stock Solutions

The reference standards stock solutions of nodifloretin (**2**), onopordin (**4**), and eupafolin (**7**) were accurately weighted and dissolved in methanol and then diluted to appropriate concentration ranges for the establishment of calibration curves. All of the stock and working standard solutions were stored at 4°C until being used for analysis.

### 2.9. Sample Preparation

The different tested samples were weighted and dissolved with M-FA (50 : 50, v/v) to a final concentration of 1 mg/ml. The sample solution was filtered through a 0.45 *μ*m membrane filter prior to injection into the HPLC system.

### 2.10. Method Validation

The analytical method was validated for linearity, limit of detection and quantification (LOD and LOQ), precision (interday and intraday), repeatability, and recovery test, following the reports on quantitative determination [18, 19]. Standard stock solutions of onopordin (**4**) and eupafolin (**7**) were prepared as shown in section 2.8 and diluted into 0.5, 1.0, 5.0, 10.0, and 15.0 *µ*g/ml of** 4**, and 1.0, 3.0, 5.0, 7.0, and 10.0 *µ*g/ml of** 7**, respectively. The calibration curve was performed by analyzing the two reference solutions in triplicate at five concentrations and drawn by plotting the peak areas versus the injection quantity of each compound. The LOD and LOQ under the present chromatographic conditions were evaluated at* S/N* of 3 and 10, respectively.

### 2.11. Data Analysis

In the bioassay, the average values of three independent analyses were presented as means ± S.D. In the chromatographic fingerprint, data analysis calculated the correlative coefficient for samples and compared the similarities of different chromatograms with the mean chromatogram among the samples tested.

## 3. Results and Discussion

### 3.1. Free Radicals Scavenging and Tyrosinase Inhibitory Activities

Bioassay-guided fractionation of the PNM led to the isolation of nine flavonoids, 3,7,4′,5′-tetrahydroxy-3′-methoxyflavone (**1**), nodifloretin (**2**), 4′-hydroxywogonin (**3**), onopordin (**4**), cirsiliol (**5**), 5,7,8,4′-tetrahydroxy-3′-methoxyflavone (**6**), eupafolin (**7**), hispidulin (**8**), and larycitrin (**9**) (Table 1).  Table 2 showed the results of these compounds on radicals scavenging and tyrosinase inhibitory effects. When compared to apigenin, a common flavone in plants, and vitamin C, a well-known antioxidant and whitening agent used in cosmetic products, eupafolin (**7**) exhibited potent effects in DPPH^∙^−, ABTS^∙+^−, and O_2_^∙−^-scavenging assays and with a concentration-dependent scavenging manner (data are not shown). Compounds** 4** and** 5** showed significant DPPH^∙^− and O_2_^∙−^-scavenging activities. Compounds with a catechol moiety in the B ring and a 2,3-double bond in conjugation with a 4-carbonyl group in the ring C show potent radical scavenging activity [20].

**Figure 1:**
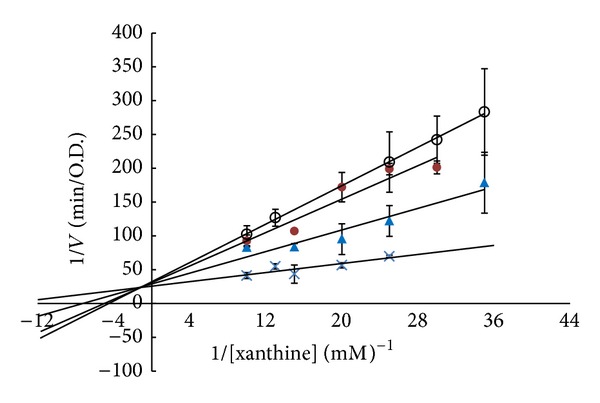
Lineweaver-Burk plot of xanthine oxidase inhibition of eupafolin (**7**) with various concentrations of xanthine. (×) Without eupafolin, (▲) 5 *μ*M of eupafolin, (●) 10 *μ*M of eupafolin, and (○) 20 *μ*M of eupafolin. Data are presented as mean ± SD of three independent experiments.

In the O_2_^∙−^-scavenging assay, eupafolin (**7**) showed strong scavenging activity with a SC_50_ value of 8.3 ± 0.4 *μ*M (Table 2).  Figure 1 showed the Lineweaver-Burk transformation of data, indicating that compound** 7** was a mixed-type inhibitor of xanthine oxidase (XO), with* Ki* values of 4.48, 5.05, and 8.93 *μ*M and *V*_max⁡_ (ΔO.D./min) of 3.6 × 10^−2^, 3.2 × 10^−2^, and 3.1 × 10^−2^ for** 7** at 5, 10, and 20 *μ*M, respectively. Biochemically, this enzyme inhibitor is associated with the hydrogen binding of phenolic hydroxyls or carbonyls of the substrate with the amide carbonyls or amino group in the peptide chain of the enzyme [21]. Compound** 7**, with a catechol moiety and a conjugated carbonyl group, may be bound to free enzymes and form an enzyme substrate complex. Thus, let compound** 7 **exhibit a potent inhibitory effect on XO activity, decreasing O_2_^∙−^ generation. The generation of free radicals and reactive oxygen species (ROS) are involved in various diseases such as hepatitis, inflammation, carcinogenesis, and aging [22]. ROS may induce melanocyte proliferation and the release of *α*-melanocyte-stimulating hormone (*α*-MSH). In this case, an excess of melanogenesis can lead to abnormal pigmentation [23]. Hence, the use of radical scavengers or antioxidants to prevent pigmentation disorders and skin aging in cosmetic and medicinal industries is becoming increasing tendency.

**Figure 2:**
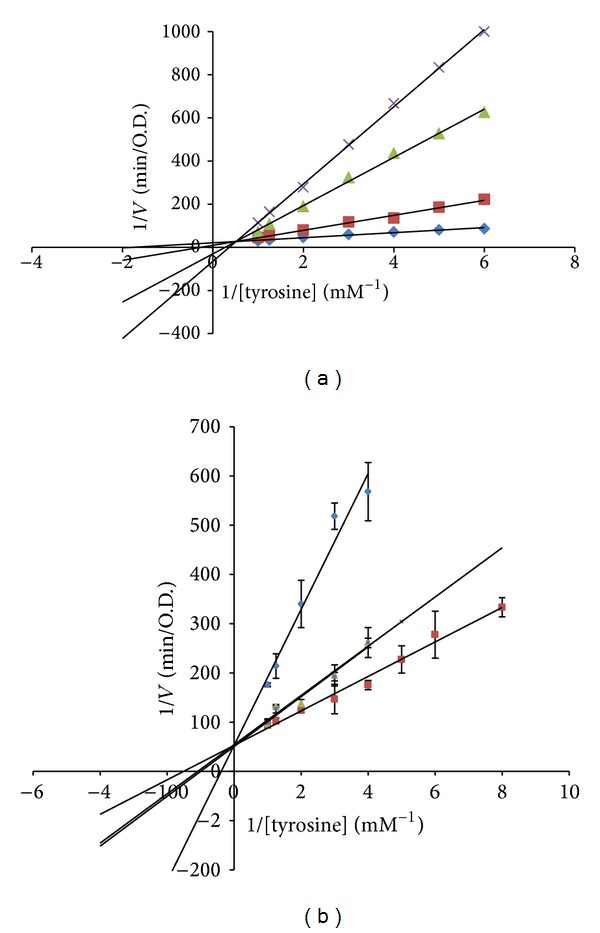
Lineweaver-Burk plot of tyrosinase inhibition of (a) onopordin (**4**) and (b) eupafolin (**7**) with various concentrations of tyrosine. (a) (◆) Without onopordin, (■) 10 *μ*M of onopordin, (▲) 60 *μ*M of onopordin, and (×) 100 *μ*M of onopordin. (b) (■) Without eupafolin, (▲) 28 *μ*M of eupafolin, (×) 56 *μ*M of eupafolin, and (◆) 112 *μ*M of eupafolin. Data are presented as mean ± SD of three independent experiments.

Many antioxidants and tyrosinase inhibitors are used in cosmetics and food products for depigmentation or antibrowning, but they have been alleged to have serious side effects. For example, vitamin C is unstable and photosensitive. High doses of arbutin induce chronic poisoning of the skin, resulting in ochronosis [24]. Kojic acid loss function after exposure to sunlight can cause contact allergy after usage [25]. Seeking new and safe depigmentation agents from natural products has been noticed in the past few years. Numerous flavonoids have been reported to be relatively mild and safe tyrosinase or melanogenesis inhibitors, whereas they exhibit antioxidant, anti-inflammatory, and other biological activities [19, 26, 27]. Since onopordin (**4**), cirsiliol (**5**), and eupafolin (**7**) displayed a potent antioxidant ability, they probably also possess good tyrosinase inhibitory effect. Thus, this study also evaluated the antityrosinase activity of the isolates of PNM.  Figure 2 and Table 2 showed that onopordin (**4**), with an IC_50_ value of 65.8 ± 3.2 *μ*M, acted as a mixed-type inhibitor, while eupafolin (**7**) acted as a competitive inhibitor and strongly inhibited the activity of mushroom tyrosinase, with an IC_50_ value of 56.0 ± 5.0 *μ*M. Tyrosinase is able to use mono-, di-, and trihydroxyphenols as substrates; among these, dihydroxyphenols (catechols) show the maximum activity, indicating that the enzyme is most active with catechol as a substrate [28, 29]. Also, the catechol structure has been reported to bind copper ions and thus may compete with tyrosinase for the available copper ions [30], leading to tyrosinase dysfunction. This suggests that a catechol moiety in the B ring attached to an *α*,*β*-unsaturated carbonyl group forms a skeleton similar to that of l-Dopa. Therefore, compounds** 4** and** 7** might bind to the binuclear active site of the enzyme and compete with tyrosine and l-Dopa as a substrate. The replacement of the substrate and the inhibition of tyrosinase activity of compounds** 4** and** 7** might inhibit the melanogenesis. Thus, onopordin (**4**) and eupafolin (**7**) with good antioxidant and antityrosinase effects can be considered candidates for cosmetic or therapeutic purposes in humans for aging and hyperpigmentation treatments. However, further investigations are required to determine their mechanisms of action.

### 3.2. Optimization of HPLC Conditions

To obtain the chemical information and valid chromatographic conditions, the columns, mobile phase compositions, detection wavelength, and gradient elution procedure were investigated in this study.

Two kinds of reverse-phase columns, LiChroCART 250-4 C18 column (250 mm × 4.6 mm i.d., 5 *μ*m) and Hypersil ODS-C18 column (250 mm × 4.6 mm i.d., 5 *μ*m), were investigated; the Hypersil ODS-C18 column was found to be more suitable and gave good peak separation and sharp peaks.

The effect of mobile phase compositions (acetonitrile (ACN)/0.1% FA, M/0.1% FA, and M/0.1% acetic acid (AA)) on chromatographic separation was investigated and it was found that there was no obviously distinction between ACN/0.1% FA, M/0.1% FA, and M/0.1% AA. While adding 0.1% AA, some peaks overlapped and the resolution of separation was slightly decreased. Considering the high-toxicity and price of ACN and the better separation condition, the binary mixture of M/0.1% FA was chosen for gradient elution.

**Figure 3:**
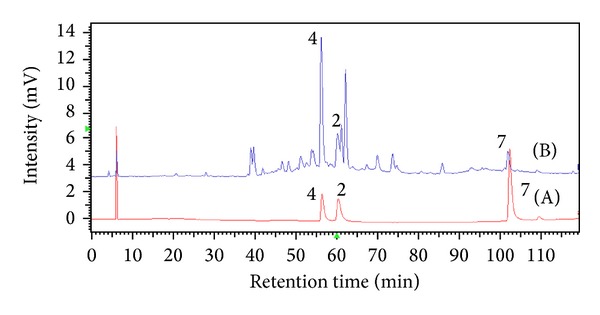
(A) The chromatogram of mixture standard compounds: nodifloretin (**2**), onopordin (**4**), and eupafolin (**7**). (B) Chromatographic fingerprint of PNM (2010-06-PNM) by HPLC-UV at 326 nm.

The wavelength for the detection of PNM and the active constituents in this plant was selected by the UV-Vis (200–400 nm) detector. Due to a full-scan experiment of PNM, its UV spectrum showed maximum adsorption at the wavelengths of 210, 273, and 326 nm. The chromatographs monitored at 210 and 273 nm revealed more peaks than at 326 nm within 15–20 min. However, due to the fact that some peaks were missing between 40 and 70 min and due to the presence of serious baseline noise, some peaks seen at 210 and 273 nm were not well separated. Meanwhile, signals response for flavones and flavonols is commonly referred to as band I (usually 300–380 nm) and band II (usually 240–280 nm). In order to detect more common peaks while achieving suitable detection of PNM and active constituents, 326 nm was selected as the most appropriate detection wavelength. The HPLC chromatograms of standards and PNM are shown in Figure 3.

### 3.3. Method Validation of Quantitative Analysis

Linearity was examined with standard solutions. According to the antioxidant and antityrosinase assay (Table 2), nodifloretin (**2**), onopordin (**4**), cirsiliol (**5**), and eupafolin (**7**) are the major active compounds, but** 5** was in a small amount and** 2** was not well separated from the unknown side-peak in the tested sample; thus, only compounds** 4** and** 7** were selected as the representative markers in this study. Each calibration curve contained five different concentrations and was performed in triplicate. The linearity for each compound was established by plotting the peak area (*y*) versus concentration (*x*) by the external standard method. The limits of detection (LOD) and quantification (LOQ) were determined at signal-to-noise ratios (*S/N*) of 3 and 10, respectively. The results are shown in Table 3.

Intra- and interday variability were utilized to evaluate precision. The mixed standard solution at low, medium, and high concentrations in one day (*n* = 3) and on three consecutive days was analyzed, respectively. The results (Table 4) indicated that the mean intraday R. S. D. values of compounds** 4** and** 7** were less than 3.97% and the mean interday R. S. D. values of compounds** 4** and** 7** were less than 6.99%.

Repeatability of this method was assessed by analyzing five independently prepared samples (2010-06-PNM). R. S. D. values of content and retention time of compounds** 4** and** 7 **were all less than 2.2%, which indicated good repeatability.

The recovery test was evaluated by standard addition method. Onopordin (**4**, 0.20 mg/g) and eupafolin (**7**, 0.15 mg/g) were spiked into the sample (2010-06-PNM) and then extracted and analyzed using the proposed procedure. As shown in Table 5, the recoveries of compounds** 4** and** 7 **were in the range of 93.50–97.33% with an R. S. D. value less than 2.40%. Therefore, this HPLC-UV method was considered precise, accurate, and sensitive enough for the quantitative evaluation of active compounds** 4** and** 7 **in* P. nodiflora.*

### 3.4. Sample Analysis

**Figure 4:**
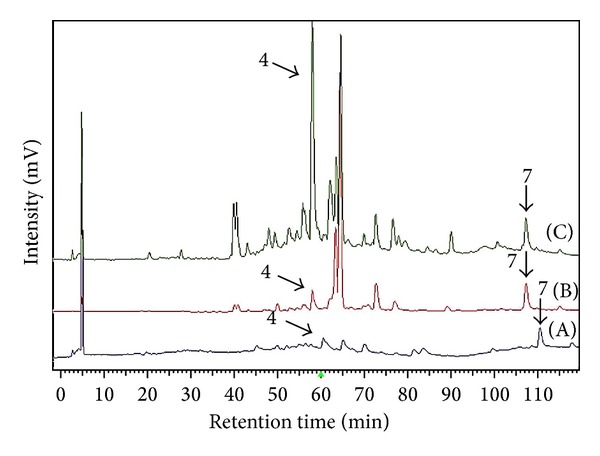
The HPLC-fingerprints of three extracts of PNM from different manufactures. (A) 2007-02-PNM; (B) 2009-06-PNM; (C) 2010-06-PNM.

The established analytical method was applied for the quantitative analysis of two active components,** 4** and** 7**, in three different batches of the aerial part of* P. nodiflora* collected at the same location in different years and seasons (Figure 4). Each sample was analyzed in triplicate to determine the mean content, and the peaks in chromatograms were identified by comparing the retention times and the online UV spectra with those of the standards. The contents of compounds** 4** and** 7** were 0.048 and 0.072 mg/g in 2007-02-PNM, 0.296 and 0.474 mg/g in 2009-06-PNM, and 0.545 and 0.381 mg/g in 2010-06-PNM, respectively. The quantitative analysis results showed that the crude extract of PNM, even in the different seasons (spring and summer), generally contained the two selected active constituents. From the results obtained, the contents of active compounds were higher in the summer than in the spring. It is known that the different growing place, climate, and harvest processing may affect the level of these compounds. Although PNM in the autumn and in the winter was not collected and detected, the results showed PNM in blossom season (May to August, summer), yielding more active compounds, might serve the good quality and quantity of PNM when used as a cosmetic or herbal medicine additive.

## 4. Conclusions

A HPLC-UV chromatographic fingerprint analysis of* P. nodiflora* was established. The antioxidant assay and the chromatogram profile of this plant could provide information about the main significant secondary metabolites represented by onopordin (**4**) and eupafolin (**7**). The results indicated that the contents of active constituents varied greatly among the PNM samples collected in different periods, with the total contents of active constituents being higher in PNM collected in the summer. This could be helpful for further evaluation of the quality of PNM.

## Figures and Tables

**Table 1:** Structure of compounds** 1**–**9** of *P.  nodiflora*.

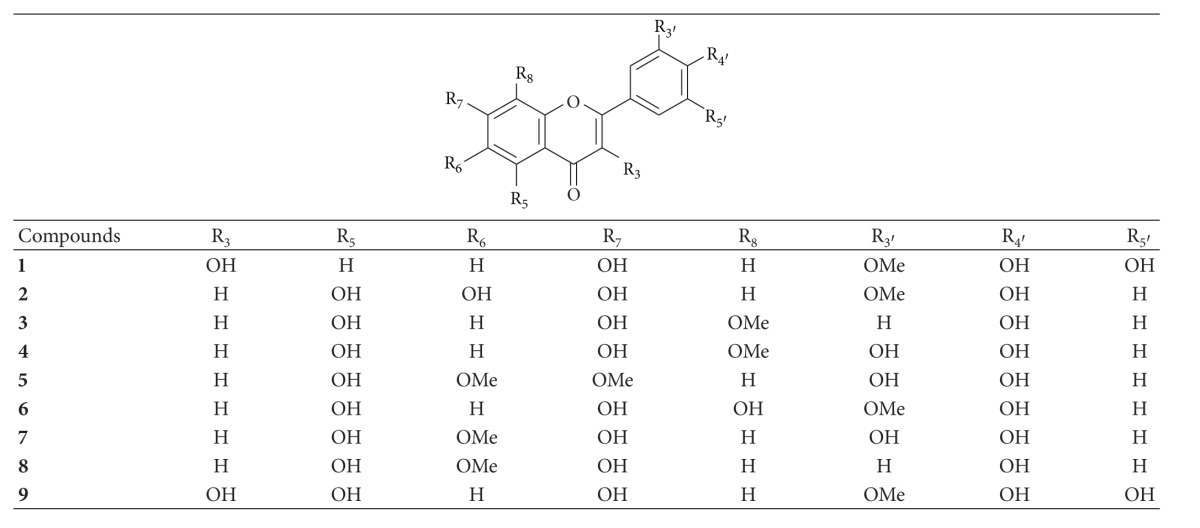

**Table 2:** Antioxidant and tyrosinase inhibitory activities of compounds **1–9** isolated from* P. nodiflora* ^a^.

Samples	DPPH^∙^	ABTS^∙+^	O_2_^∙−^	Tyrosinase
	SC_50_^c^ (*μ*M)			IC_50_^d^ (*μ*M)
**1**	—^e^	—^e^	—^e^	—^e^
**2**	198.2 ± 3.8	62.2 ± 2.5	42.3 ± 1.5	127.6 ± 1.3
**3**	>300	>300	59.9 ± 2.1	169.9 ± 3.8
**4**	24.7 ± 1.2	53.7 ± 3.9	23.5 ± 0.5	65.8 ± 3.2
**5**	21.8 ± 1.2	58.4 ± 2.7	10.7 ± 1.3	121.8 ± 4.8
**6**	>300	>300	34.6 ± 2.4	152.8 ± 4.4
**7**	24.1 ± 0.4	30.5 ± 1.2	8.3 ± 0.4	56.0 ± 5.0
**8**	57.5 ± 2.7	55.3 ± 3.8		146.0 ± 2.2
**9**	—^e^	—^e^	—^e^	—^e^
Apigenin^b,+^	>300	159.6 ± 4.4	81.3 ± 2.0	304.8 ± 3.1
Vit. C^b^	64.6 ± 4.4	49.9 ± 2.1	23.5 ± 1.7	—^e^
Kojic acid^b^	—^e^	—^e^	—^e^	88.1 ± 1.8

^a^Values are presented as means ± SD (*n* = 3). ^b^Apigenin, Vit. C, and Kojic acid are used as positive controls. ^+^The data of apigenin are referred to previous report (Lan et al. 2013) [15]. ^c^SC_50_ is the concentration of test samples that scavenges 50% radicals. ^d^IC_50_ is the concentration of test samples that inhibits 50% tyrosinase. ^e^Not tested.

**Table 3:** Calibration curves, linear ranges, and LODs and LOQs of onopordin (**4**) and eupafolin (**7**) by HPLC.

Analytes	Calibration curve	linear range (*µ*g/mL)	*R* ^2^ (*n* = 3)	LOD (*µ*g/mL)	LOQ (*µ*g/mL)
**4**	*y* = 509933*x* − 1863.7	0.50–15.0	0.9999	0.05	0.26
**7**	*y* = 62667*x* + 4543.5	1.0–10.0	0.9994	0.01	0.11

**Table 4:** Analytical results of intra- and interday variability of onopordin (**4**) and eupafolin (**7**).

Analytes	Precision (*n* = 3)		
	Concentration (*μ*g/mL)	Intraday R.S.D. (%)	Interday R.S.D. (%)
**4**	0.8	2.34	1.29
	6	2.58	4.68
	12	2.89	1.30

**7**	2	2.31	6.99
	4.5	3.49	1.50
	8.5	3.97	2.95

**Table 5:** Recoveries of onopordin (**4**) and eupafolin (**7**) in the aerial part of *P. nodiflora* (2010-06-PNM) (*n* = 3).

Analytes	Original (mg/g)	Spiked (mg/g)	Found (mg/g)	Recovery (%)	R.S.D (%)
**4**	0.545	0.200	0.732	93.50	2.40
**7**	0.381	0.150	0.527	97.33	0.91
